# STAT3 Activity Promotes Programmed-Death Ligand 1 Expression and Suppresses Immune Responses in Breast Cancer

**DOI:** 10.3390/cancers11101479

**Published:** 2019-10-01

**Authors:** Ioannis Zerdes, Majken Wallerius, Emmanouil G. Sifakis, Tatjana Wallmann, Stina Betts, Margarita Bartish, Nikolaos Tsesmetzis, Nicholas P. Tobin, Christos Coucoravas, Jonas Bergh, George Z. Rassidakis, Charlotte Rolny, Theodoros Foukakis

**Affiliations:** 1 Department of Oncology-Pathology, Karolinska Institutet, 17164 Stockholm, Sweden; majken.wallerius@gmail.com (M.W.); emmanouil.sifakis@ki.se (E.G.S.); tatjana_wallmann@web.de (T.W.); stina.betts@stud.ki.se (S.B.); margarita.bartish@ki.se (M.B.); nick.tobin@ki.se (N.P.T.); jonas.bergh@ki.se (J.B.); georgios.rassidakis@ki.se (G.Z.R.); charlotte.rolny@ki.se (C.R.); 2 Department of Women’s and Children’s Health, Karolinska Institutet, 17177 Stockholm, Sweden; nikolaos.tsesmetzis@ki.se; 3 Department of Medical Biochemistry and Biophysics, Karolinska Institutet, 17165 Stockholm, Sweden; christos.coucoravas@ki.se; 4 Breast Center, Theme Cancer, Karolinska University Hospital, 17176 Stockholm, Sweden; 5 Department of Pathology and Cytology, Karolinska University Hospital, 17176 Stockholm, Sweden

**Keywords:** breast cancer, PD-L1, STAT3, M2 macrophages, NK cells, STAT3 inhibitor XIII

## Abstract

Signal transducer and activator of transcription 3 (STAT3) is an oncogene and multifaceted transcription factor involved in multiple cellular functions. Its role in modifying anti-tumor immunity has been recently recognized. In this study, the biologic effects of STAT3 on immune checkpoint expression and anti-tumor responses were investigated in breast cancer (BC). A transcriptional signature of phosphorylated STAT3 was positively correlated with PD-L1 expression in two independent cohorts of early BC. Pharmacologic inhibition and gene silencing of *STAT3* led to decreased Programmed Death Ligand 1 (PD-L1) expression levels in vitro, and resulted as well in reduction of tumor growth and decreased metastatic dissemination in a mammary carcinoma mouse model. The hampering of tumor progression was correlated to an anti-tumoral macrophage phenotype and accumulation of natural-killer cells, but also in reduced accrual of cytotoxic lymphocytes. In human BC, pro-tumoral macrophages correlated to PD-L1 expression, proliferation status and higher grade of malignancy, indicating a subset of patients with immunosuppressive properties. In conclusion, this study provides evidence for STAT3-mediated regulation of PD-L1 and modulation of immune microenvironment in BC.

## 1. Introduction

Programmed Death 1 (PD-1, *CD279*) and its ligand Programmed Death Ligand 1 (PD-L1, *CD274*) are transmembrane proteins with role in autoimmunity, infection and anti-tumor immune response. PD-L1 is mostly expressed in tumor cells but also in dendritic cells and macrophages, while its receptor PD-1 is predominantly expressed in activated T-cells [1]. Their engagement leads to T-cell inactivation and to impairment of effective immune response against the tumor [2]. Therefore, the PD-1/PD-L1 axis represents an important immune checkpoint, and its targeting with monoclonal antibodies has been proven to be an effective immunotherapeutic strategy, demonstrating durable clinical responses and improved survival in several tumor types [3].

Breast cancer has been considered as a relatively non-immunogenic tumor due its low mutational burden. However, the presence of tumor-infiltrating lymphocytes has demonstrated prognostic and predictive value—at least in the triple negative and HER2 positive subtypes [4,5]. Data indicating the efficacy of immune checkpoint blockade in breast cancer patients are mostly derived from phase I and II clinical trial results [6], and recently the first phase III trial results showed that the addition of the anti-PD-L1 antibody atezolizumab to nab-paclitaxel was associated with improved progression-free survival in patients with triple-negative metastatic breast cancer [7].

Various genetic, transcriptional and post-translational factors have been involved in the regulation of PD-L1 and these may be tumor type-specific [8]. Among them, signal transducer and activator of transcription 3 (STAT3) represents a crucial transcription factor for cell proliferation, survival and tumor development [9]. A direct link between STAT3 and PD-L1 expression has been previously described [10], and recent data have provided insight into this regulatory mechanism [11]. STAT3 mediates the expression of important regulators of cell cycle and apoptosis but it can also play an important role in tumor-immune cells interaction by impairing effective antitumor immunity [12]. STAT3-mediated release of various cytokines and chemokines can interact and influence components of the tumor microenvironment (TME) and especially immune cell accumulation including T-cells, Natural Killer (NK) cells as well as tumor-associated macrophages (TAMs) [13]. Of note, TAMs represent a heterogeneous subpopulation with either pro-tumoral or anti-tumoral properties. Their accumulation has been correlated with a worse prognosis and therapeutic resistance in most solid tumors, including breast cancer [14].

In the present study, the role of STAT3 in the regulation of PD-L1 expression and in the potential modifications of the immune microenvironment in breast cancer was investigated. Our findings provide evidence for STAT3-mediated regulation of PD-L1 *in vitro* and impact on accumulation of pro-tumoral macrophages and other immune cell subpopulations in an *in vivo* murine mammary tumor model. The interactions of PD-L1 with STAT3, pro-tumoral macrophages and tumor characteristics have been explored as well in a well-characterized cohort of breast cancer patients.

## 2. Results

### 2.1. Association Between PD-L1 and STAT3 Expression in Breast Cancer Cell Lines and Human Breast Cancer

**Figure 1:**
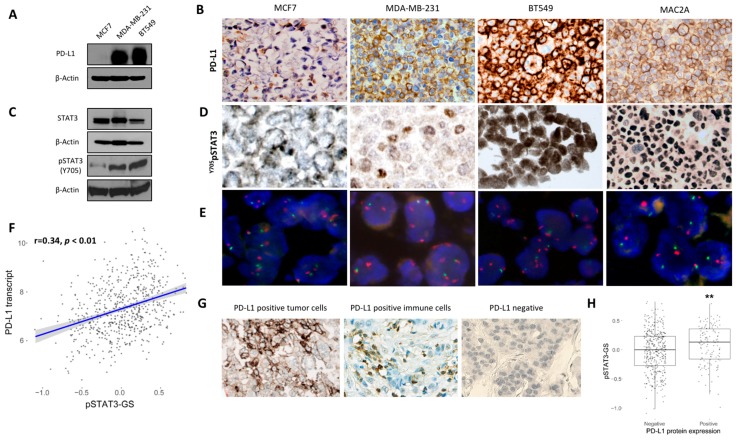
Expression of PD-L1 and STAT3 in breast cancer cell lines and in breast cancer patients. (**A**) Expression of PD-L1 protein in breast cancer cell lines in immunoblots. (**B**) Immunohistochemical expression of PD-L1 in breast cancer cell lines using FFPE cell blocks. (**C**) Protein expression of STAT3 and STAT3 phosphorylation at Tyr705 (Y705) residue in immunoblots in breast cancer cell lines. (**D**) Immunohistochemical expression pSTAT3 (Y705) in breast cancer cell lines using FFPE cell blocks. The anaplastic large cell lymphoma cell line Mac2A was used as a positive control. Original magnification: 400×. (**E**) Fluorescence in situ hybridization (FISH) analysis for PD-L1 probe performed on FFPE cell blocks. The validated probe (green signal) covers the gene locus at 9p24.1. A centromeric chromosome 9 probe (CEN9, red signal) was used as a control. No *PD-L1* gene amplification was demonstrated in Mac2A cell line. Original magnification: 630×. (**F**) Correlation of *PD-L1* transcript expression with a previously published pSTAT3-associated gene signature (pSTAT3-GS) reflecting the status of pSTAT3 expression in breast cancer patients with available gene expression profiling data (*n* = 619). (**G**) PD-L1 expression was evaluated in human breast cancer samples using immunohistochemistry in tissue microarrays. Representative patient cases with positive expression in tumor cells (left panel), immune cells (middle panel) and negative expression (right panel) are shown. Original magnification: 400×. (**H**) Correlation between samples with positive and negative PD-L1 protein expression on either tumor or immune cells (total cells) with pSTAT3-GS in breast cancer patients (*n* = 539, Wilcoxon-Mann-Whitney test, *p* = 0.0027, ** *p* < 0.01 ).

The association between PD-L1 and STAT3 expression was first assessed in human breast cancer cell lines. PD-L1 expression pattern was evaluated in three different human breast cancer cell lines by western blot analysis and immunohistochemistry. MDA-MB-231 and BT549 demonstrated high levels of PD-L1 compared to MCF7 cells, which showed very low—almost undetectable—PD-L1 protein (Figure 1A,B). STAT3 showed a ubiquitous expression in all three cell lines, as visualized by western blot (Figure 1C), while MDA-MB-231 and BT549 cells showed a higher degree of STAT3 phosphorylation (at the Y705 residue) compared to MCF7, as visualized by western blot (Figure 1C) and immunohistochemistry (Figure 1D), notably following the pattern of PD-L1 expression (Figure 1A,B). Importantly, no amplification of the *PDL1* gene locus was detected in any of the breast cancer cell lines as assessed by Fluorescence In Situ Hybridization (FISH) analysis performed in sections of FFPE cell blocks (Figure 1E). The association between pSTAT3 and PD-L1 was further assessed in a human breast cancer cohort for which primary tumor gene expression data were available (*n* = 619) and PD-L1 protein levels were assessed (*n* = 539). The scores of a previously published metagene signature of pSTAT3 in breast cancer (*pSTAT3-*GS) were positively correlated with *PD-L1* transcript expression levels (Spearman’s rho = 0.34; *p* < 2.2e−16) (Figure 1F). The positive association between pSTAT3-GS score and PD-L1 transcript was also confirmed when RNA-sequencing data derived from the Cancer Genome Atlas (TCGA) Provisional dataset (*n* = 1081) (Spearman’s rho = 0.38, *p* < 0.01; Supplementary Figure S2A) were used. Additionally, in positive cases for PD-L1, total cell protein expression was evaluated by immunohistochemistry (Figure 1G), and *pSTAT3*-GS scores were significantly higher than those in the PD-L1 negative cases (*p* = 0.0027) (Figure 1H). Moreover, in a subset of patients (*n* = 83) pSTAT3 was assessed by IHC, and high pSTAT3 protein levels were positively associated with PD-L1—especially in immune cells (Figure S3, Table S4).

**Figure 2:**
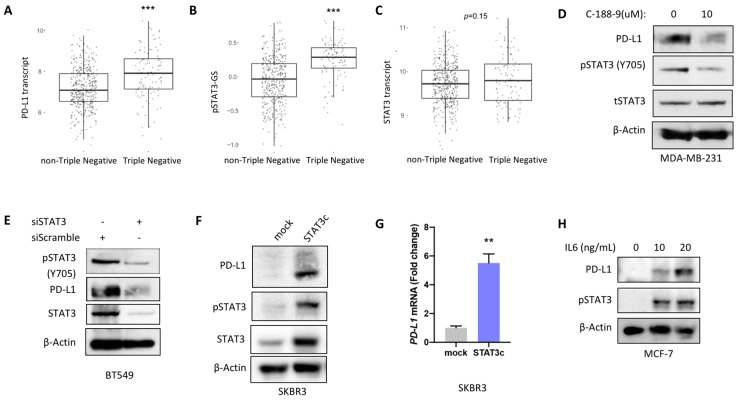
Expression patterns of STAT3 and PD-L1 in human breast cancer subtypes and regulation of PD-L1 by STAT3 in breast cancer cell lines. (**A**) Expression levels of *PD-L1* transcript, (**B**) pSTAT3-GS score and (**C**) STAT3 transcript in triple-negative versus non-triple negative breast cancer patients. (**D**) Inhibition of STAT3 activity by using STAT3 inhibitor C-188-9 (XIII) resulted in decreased levels of PD-L1 expression in immunoblot 48 h following treatment in MDA-MB-231 breast cancer cell line. (**E**) Knocking down *STAT3* using specific siRNA construct led to decreased levels of PD-L1 in the transiently transfected BT549 cell line. Stable transfection of SKBR3 breast cancer cell line with a STAT3 overexpressing plasmid (STAT3c) resulted in increased levels of PD-L1 (**F**) protein and (**G**) transcript expression. qPCR data are illustrated as the fold change relative to control and normalized to β-actin. They represent one out of three independent experiments and are depicted as the mean (±standard error of the mean, SEM; ** *p* < 0.01). (**H**) Increased protein levels of PD-L1 were noted in MCF7 cell line in response to treatment with IL-6.

Additionally, the expression of PD-L1 transcript and pSTAT3-GS score were higher in triple-negative (TN) versus non-TN breast tumors in both cohorts (Figure 2A,B and Figure S2) whereas total STAT3 gene expression did not differ between these two groups (Figure 2C and Figure S2).

### 2.2. STAT3 Mediated Regulation of PD-L1 Expression In Vitro

Next, STAT3 regulation of PD-L1 expression was investigated using the STAT3 selective inhibitor C188-9 (XIII). Suppression of pSTAT3 activity decreased PD-L1 protein levels in MDA-MB-231 cell line, as detected by western blots (Figure 2D). Additionally, silencing *STAT3* gene expression using specific siRNA constructs led to decreased PD-L1 protein levels in transiently transfected BT549 cells (Figure 2E). Furthermore, PD-L1 expression in the SKBR3 breast cancer cell line stably transfected with a constitutively active STAT3 construct was examined. Constitutive activation of STAT3 resulted in increased protein and mRNA levels of PD-L1 (Figure 2F,G). Treatment of the PD-L1 negative BC cell line MCF7 with IL-6 resulted in increased protein levels of both PD-L1 and pSTAT3 (Figure 2H).

### 2.3. Stat3 Silencing Downregulates PD-L1 in Mouse Cells, Restricts Tumor Growth and Metastatic Formation and Modifies Anti-Tumor Immune Response In Vivo

**Figure 3:**
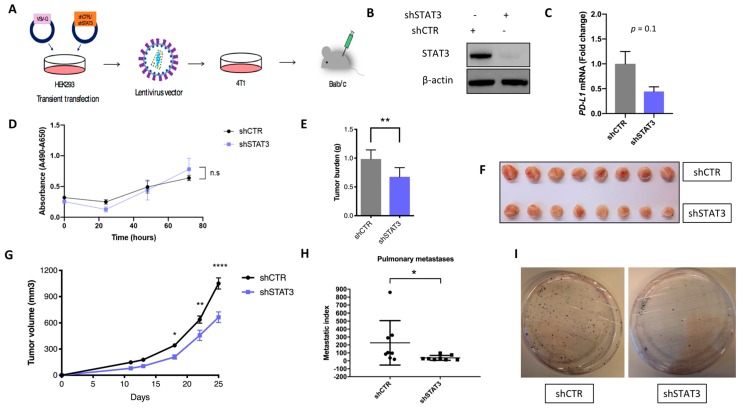
STAT3-mediated regulation of PD-L1 and effect on tumor growth and metastatic dissemination in vivo. (**A**) Schematic representation of the generation of the 4T1 breast cancer mouse model from transient transfection of HEK293 cells with shCTRL/sh*Sta3* plasmids and production of lentiviruses to transduction of 4T1 mouse breast cancer cell line, which was injected into the mammary fat pad of to Balb/c mice. (**B**) Downregulation of STAT3 protein levels in 4T1 cell line as assessed via immunoblotting upon sh*STAT3* plasmid transduction. (**C**) Decreased *pd-l1* transcript levels in sh*Stat3* 4T1 cell line as evaluated by qPCR. qPCR data are depicted as the fold change relative to control and are normalized to *18S* rRNA. Data represent one out of three independent experiments and presented as the mean ± SEM. (**D**) 4T1-shCTRL and 4T1-sh*STAT3* were cultured for three days under normal conditions and XTT proliferation assay was performed to assess cancer cell proliferation. The proliferation rate of *Stat3* silenced cells was not significantly changed compared to the control cells. Each time point denotes the mean of six replicates (±—standard deviation, SD). Two-way ANOVA test was used. (**E**–**G**) 4T1-shCTRL and 4T1-sh*Stat3* tumor cells were injected into the mammary fat pad of BALB/c mice (*n* = eight mice per group). Graphs display the tumor weight (**E**) and tumor volume (**G**). Student’s t-test and two-way ANOVA with Sidak’s multiple comparison test were used, respectively (* *p* < 0.05; ** *p* < 0.01, *** *p* < 0.001; **** *p* < 0.0001). shCTR and s*hStat3* mice tumors are depicted in picture (**F**). (**H**–**I**) The graph depicts metastatic index, which is calculated by the number of well-formed colonies per tumor weight (**H**) (Wilcoxon-Mann-Whitney test, *p* = 0.0148). Representative photos from the colongenic assay are depicted in figure (**I**).

The impact of *Stat3* silencing on PD-L1 levels was explored in a murine model of breast cancer. More specifically, knocking down *Stat3* gene using a specific shRNA plasmid in mouse mammary carcinoma 4T1 cells (sh*Stat3* cells) resulted in decreased levels of *pd-l1* (Figure 3A–C) compared to cells transduced with corresponding empty vector (shCTR cells). When injected into the mammary fat pad of BALB/c mice, sh*Stat3* tumors displayed a decreased tumor volume (Day 25: 36.7%) and tumor weight (Day 25: 31.3%) compared to shCTR tumors (Figure 3E–G). In addition, suppression of *Stat3* expression resulted in decreased pulmonary metastatic index compared to control (Figure 3H,I).

**Figure 4:**
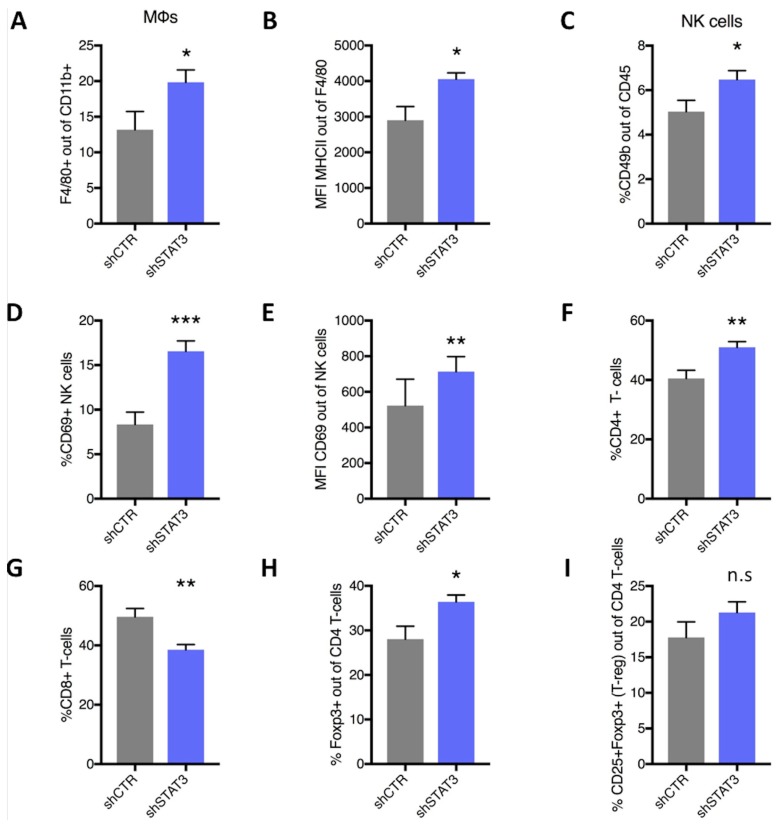
Effect of *Stat3* silencing on immunologic profile in mouse model. 4T1-shCTRL and 4T1-sh*Stat3* tumors were dissociated to a single cell level and analysed using flow cytometry for the percentage of (**A**). F4/80^+^ cells out of the gate of CD11b^+^. Graph (**B**) shows the mean fluorescence intensity (MFI) of MHCII out of F480+ cells in 4T1-shCTRL and 4T1-sh*Stat3* tumors. (**C**) Percentage of CD49b^+^ NK cells out of CD45 and percentage (**D**) and MFI (**E**) of CD69 out of NK cells in the same tumors. 4T1-shCTRL and 4T1-sh*Stat3* tumors were also analysed with flow cytometry for the percentages of CD4^+^ T-cells (**F**), CD8+ T-cells (**G**), FoxP3^+^ (**H**) and CD25^+^ FoxP3^+^ cells out of CD4^+^ T-cells (**I**). Student’s t-test was performed for all comparisons depicted in the graphs and flow cytometry data are presented as mean ± SEM (*n* = 8; * *p* < 0.05; ** *p* < 0.01; *** *p* < 0.001); MHC: major histocompatibility complex.

Furthermore, it was investigated whether the reduction in tumor progression by silencing *Stat3* expression could affect the tumor immune profile. In fact, flow cytometry analysis (Figures S5 and S6) showed that silencing *Stat3* resulted in a significant increase in F4/80^+^ TAMs (Figure 4A). However, these TAMs displayed increased levels of major histocompatibility complex class II (MHC II), indicating a more “M1”-like anti-tumoral phenotype compared to shCTR tumors (Figure 4B) [15,16]. Consistently with an anti-tumoral phenotype, NK cell accumulation was increased in sh*Stat*3 tumors compared to controls (Figure 4C). Furthermore, these NK cells displayed higher expression of CD69 (Figure 4D,E) indicating that they were more activated in sh*Stat3* tumors compared to the controls. Silencing of *Stat3* resulted also in an increase of CD4^+^ T cells (Figure 4F) while cytotoxic CD8^+^ T cells accumulation was reduced, as compared to controls (Figure 4G). These CD4^+^ T cells displayed increased expression of FoXP3^+^ in combination with CD25 (Figure 4H,I), indicating an immunosuppressive feature.

### 2.4. CD163^+^ TAM Phenotype Is Associated with Higher PD-L1 Expression, Grade and Proliferation in Breast Cancer Patients

**Figure 5:**
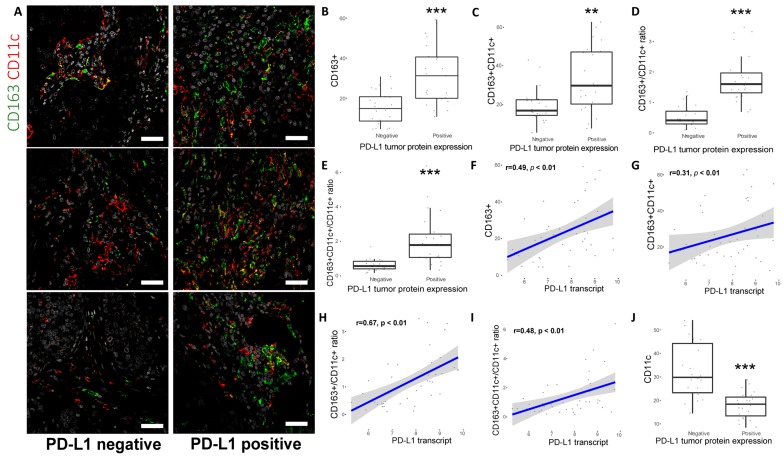
Pro-tumoral TAM phenotype correlates with PD-L1 expression levels in human breast cancer patients. (**A**) Double immunofluorescence staining with markers for antigen presenting cells such as M1-like TAMs (CD11c: red) and pro-tumoral M2-like TAMs (CD163: green) macrophages was performed in patients with PD-L1 positive (*n* = 23) and PD-L1 negative (*n* = 22) expression in tumor cells. Scale bar, 100 um. Correlations of PD-L1 protein expression in tumor cells with percentage of CD163^+^ cells (**B**), percentage of CD163^+^CD11c^+^ cells (**C**), CD163^+^/CD11c^+^ ratio and CD163^+^ CD11c+/CD11c+ ratio (M2-like subtype 2 versus M1-like phenotypes) (**D**–**E**). (**F**–**I**) The same percentages and ratios were also positively correlated with *PD-L1* transcript levels. An inverse correlation was noted between CD11c^+^ cells with PD-L1 positive tumor protein expression (**J**). Wilcoxon-Mann-Whitney test and Spearman’s rank correlation coefficient were used (* *p* < 0.05; ** *p* < 0.01; *** *p* < 0.001).

**Figure 6:**
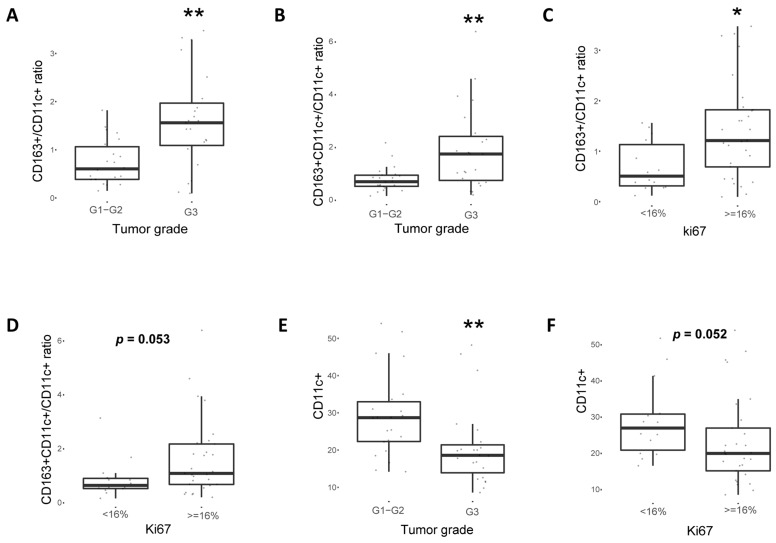
CD163^+^ macrophage phenotype correlates with higher Ki67 and grade in human breast cancer patients. CD163^+^/CD11c^+^ and CD163^+^CD11c^+^/CD11c^+^ ratios were positively correlated with high grade (**A**,**B**) and high Ki67 (**C**,**D**) in breast cancer patient patients (*n* = 45). CD11c^+^ cell expression was associated with lower tumor grade and lower Ki67 (**E**,**F**). Wilcoxon-Mann-Whitney test was used (* *p* < 0.05; ** *p* < 0.01; *** *p* < 0.001).

Apart from the mouse model, it was further explored whether PD-L1 expression correlated to a pro-tumoral “M2”-like (CD163^+^) TAM phenotype or an anti-tumoral CD11c^+^ macrophage/dendritic cell phenotype in breast cancer patient samples. Hence, 45 patient samples with PD-L1 positive (*n* = 23) or PD-L1 negative (*n* = 22) expression in tumor cells were stained for CD11c and CD163 (Figure 5A) and three different populations could be detected, i.e., CD163^+^CD11c^−^, CD163^+^CD11c^+^ and CD163^-^CD11c^+^. Of note, both the percentage of CD163^+^ and CD163^+^CD11c^+^ cells (Figure 5B,C) as well as their ratio to CD11c^+^ antigen-presenting cells (CD163^+^/CD11c^+^; Figure 5D and CD163^+^CD11c^+^/CD11c^+^; Figure 5E) were significantly higher in patients with PD-L1 positive expression in tumor cells. Similar results were observed when these subpopulations were associated with *PD-L1* transcript levels (Figure 5F–I). Conversely, higher percentage of CD11c^+^ cells was observed in patients with PD-L1 protein negative tumors (Figure 5J) and were inversely correlated with *PD-L1* mRNA as well (data not shown). Accumulation of CD163^+^CD11c^-^, CD163^+^CD11c^+^ cells (Figure S4) and their respective ratio to CD11c^+^ cells (Figure 6A,B) were observed in patients with grade 3 tumors and with high expression of the proliferation marker Ki67 (Figure 6C,D and Figure S4). In contrast, CD11c^+^ were more prominent in patients with grade 1–2 tumors and with low expression of the proliferation marker Ki67 (Figure 6E,F). In corroborration, PD-L1 tumor IHC expression was significantly associated with high grade and Ki67 expression (Table S5).

## 3. Discussion

Immune checkpoint blockade has revolutionized cancer treatment, improving survival outcomes in cancer patients, but the underlying mechanisms of PD-L1 regulation are not yet fully understood [8]. Activated (phosphorylated) STAT3, which forms dimers and transports into the nucleus, represents a key transcription factor that critically controls proliferation, invasiveness, survival and metastasis [17]. It also modifies the immune response through various mechanisms, including regulation of PD-L1 expression [18]. Here, it was shown that STAT3 regulated PD-L1 expression in breast cancer cell lines and its silencing led to restriction of tumor growth and altered the immune profile in a murine breast cancer model. Moreover, PD-L1 expression was associated with a pro-tumoral TAM phenotype in breast cancer patients.

Even though a direct link between PD-L1 and STAT3 has been described in previous reports, in this study it was shown that STAT3 can influence immune response and PD-L1 expression in mouse models as well as in patients with early breast cancer. As indicated in a previous breast cancer cell line study, pSTAT1-pSTAT3 dimers bound on *PD-L1* gene promoter inducing its expression and therefore STAT3 inhibition led to partial downregulation of PD-L1 [19]. Similar studies suggesting a STAT3-mediated transcriptional regulation of PD-L1 have been performed in nucleophosmin—anaplastic lymphoma kinase (NPM-ALK) positive (+) anaplastic large cell lymphoma (ALCL) [10], in ALK (-) ALCL [11], in KRAS- and EGFR- mutant non-small cell lung cancer [20,21] and in head and neck squamous cell carcinoma [22]. By contrast, STAT3 was not directly bound on *PD-L1* gene promoter in melanoma cells [23]. In this study, it was confirmed that STAT3 can regulate PD-L1 expression in vitro.

STAT3 signaling contributes as well to a dynamic crosstalk between tumor cells and immune cells, including macrophages, CD8^+^ T-cells, myeloid-derived suppressor cells, T-regs and NK cells [24]. In this study, it was demonstrated that *Stat3* gene silencing in 4T1 mouse cell line not only led to tumor growth restriction but also altered the immunologic profile in vivo. Importantly, a prominent anti-tumoral macrophage phenotype was denoted in sh*Stat3* tumors compared to shCTR. Decreased lung metastases were also observed in those tumors, further underscoring the role of skewing macrophage polarization in metastatic potential [25]. Similarly to our experiments, depletion of *Stat3* in epithelial cells of transgenic PyMT-MMTV mice resulted in decreased tumor growth and metastatic potential, and macrophage accumulation in *Stat3*-deficient mice [26]. However, in our experimental setting we could also show that TAMs had acquired an “M1”-like anti-tumoral phenotype that was correlated to accumulation of activated NK cells. Surprisingly, we also observed an accumulation of immunosupressive T-regs and less cytotoxic CD8^+^ T-cells in sh*Stat3* tumors. This effect on T-cell activation could potentially be explained by the fact that PD-L1 is not only expressed on tumor cells, but also on immune cells—including macrophages. Indeed, approximately 50 % of TAMs express PD-L1 (data not shown) independent of *Stat3* expression in the 4T1 mammary tumor model system, but it is still unclear how this can influence the anti-tumor immunity. The expression of other co-inhibitory markers, transcriptional or post-transcriptional factors may also contribute to this restrained immune response. Increased PD-1 expression in TAMs was associated with impaired antitumor immune response in colorectal cancer patients [27], while in another study, PD-L1 expression in macrophages hindered their proliferation and activation [28]. On the other side, activated NK cells were elevated in sh*Stat3* tumors, indicating an anti-tumor immune activity, most likely responsible for the moderate effect on tumor growth. Of importance, the skewing of TAMs towards an anti-tumoral phenotype may instead dictate the metastatic dissemination, as these macrophages have previously been shown to hamper tumor cell extravasation into the blood vessels and form secondary tumors [25,29].

At the patient level, correlations between pSTAT3 and PD-L1 both at the mRNA and protein level were explored and demonstrated in a large breast cancer cohort. Of note, in a subset of patients PD-L1 expression in tumor cells was significantly associated with “M2”-like macrophage phenotype (driven by the expression of CD163) while both PD-L1 and “M2”-like macrophages were associated with higher Ki67 expression and tumor grade. Although it has long been described that TAM accumulation is correlated with tumor progression and a worse prognosis [30], recent reports featured their interaction with immune checkpoints in tumor cells and in TME components [31]. Specifically, the two distinct macrophage phenotypes, the anti-tumoral “M1”-like and the pro-tumoral “M2”-like can secrete cytokines and other factors, which in turn affect PD-L1 expression and anti-tumor immune response [32,33] and only few recent studies have shown correlation of PD-L1 levels in tumor cells with macrophages phenotypes. In a study of gastric adenocarcinoma “M2”-like macrophage infiltration was correlated with PD-L1 expression [34] while in a mouse model, anti-PD-1 therapy led to macrophage reprogramming from “M2”-like to “M1”-like phenotype and to a subsequent regression of osteosarcoma lung metastases [35].

The findings of this study have potential clinical implications. First, therapeutic strategies involving STAT3 inhibition could enhance the efficacy of anti-PD-L1/PD-1 monoclonal antibodies, which recently proved efficacy in patients with metastatic triple negative breast cancer [7]. The STAT3 SH2 domain binder inhibitor C188-9 inhibitor is currently tested in an ongoing phase I trial (NCT03195699) in patients with advanced-stage cancers, including breast cancer. Previous research findings indicated that combined targeting of IL-6/JAK/STAT pathway and PD-L1 resulted in restricted tumor growth in in vivo models [36,37], while in others, STAT3 targeting increased the efficacy of anti-PD1 mAb [38]. Currently, a phase II trial testing the combination of a pSTAT3 inhibitor (napabucasin) with the anti-PD-1 antibody nivolumab in microsatellite stable, refractory colorectal cancer (NCT03647839) is ongoing. Moreover, immune checkpoint blockade could be combined with agents (e.g., CSF-1 inhibitors) that can inhibit TAMs accumulation and/or re-programme macrophages towards a more anti-tumoral M1-like phenotype in selected breast cancer patients. Indeed, such therapeutic combinations are currently under investigation in clinical trials [31,39]. Nevertheless, the recognition of such immunosuppressive expression patterns may pave the way for the development of biomarkers and patient stratification. Hence, subgroups of patients who may gain the most benefit of conventional, targeted and/or immune therapy can be identified towards a more personalized cancer treatment approach, yet the importance of these findings still need to be prospectively investigated in breast cancer patients.

## 4. Materials and Methods 

### 4.1. Cell Lines, Plasmids, and Reagents

The cell lines used in this study, along with their characteristics, are listed in Table S1. The human breast cancer cell lines BT549 and SKBR3, mouse mammary carcinoma cell line 4T1, as well as the control anaplastic large cell lymphoma (Mac2A) and Hodgkin lymphoma (HDLM2) cell lines were grown in complete RPMI-1640 medium (Gibco/Life Technologies, Waltham, MA, USA) supplemented with 10% fetal bovine serum (FBS) (Gibco/Life Technologies, Waltham, MA, USA), 1% L-glutamine (Gibco/Life Technologies, Waltham, MA, USA) and 1% penicillin/streptomycin (Gibco/Life Technologies, Waltham, MA, USA). The breast cancer cell lines MCF7 and MDA-MB-231 were grown in complete DMEM medium (Gibco/Life Technologies, Waltham, MA, USA) supplemented with 10% FBS, 1% L-glutamine, and 1% penicillin/streptomycin. Cells were cultured at 37 °C in 5% CO_2_. Vector control and STAT3C-transfected SKBR3 cells were obtained from Sarah Walker and David Frank, Department of Medical Oncology, Dana-Farber Cancer Institute, and Department of Medicine, Harvard Medical School, Boston, MA, USA. Breast cancer cells were treated with STAT3 inhibitor C188-9/XIII (Calbiochem, St. Louis, MO, USA) and with recombinant human IL-6 (PeproTech, Rocky Hill, NJ, USA) at the indicated concentrations for 48 and 24 h respectively and whole cell lysates were prepared for western blot analysis.

### 4.2. Western Blot Analysis

Tumor cells were collected, washed in cold phosphate-buffered saline (PBS) (GE Healthcare Life Sciences, Chicago, IL, USA), lysed in lysis buffer and western blot analysis was performed as previously described [40]. The antibodies used in the present study are listed in Table S2.

### 4.3. RNA Extraction, cDNA Synthesis, and Real Time Quantitative Polymerase Chain Reaction (RT-qPCR)

Total RNA was extracted with the RNeasy^®^ Plus Mini Kit (QIAGEN Inc., Hilden, Germany) and cDNA was synthesized using the Superscript First Strand Synthesis System (Invitrogen Life Technologies, Carlsbad, CA, USA) according to the manufacturer’s protocol. The mRNA expression levels were quantified by RT-qPCR using the Power SYBR^®^ Green PCR Master Mix (Applied Biosystems by Thermo Fisher Scientific, Waltham, MA, USA) in a one-step reaction and the mRNA expression levels were determined by the comparative CT (ΔΔCt) method. 18S rRNA and beta-actin were used as the endogenous control genes as indicated. The primer sequences used are listed in Table S3. The RT-qPCR program included Amplitaq Gold DNA polymerase activation at 95 °C (10 min) followed by 40 cycles of DNA denaturation (95 °C for 15 sec) and annealing/extension (60 °C for 30 sec). All reactions were performed using a Veriti 96-well thermal cycler (Applied Biosystems by Thermo Fisher Scientific, Waltham, MA, USA).

### 4.4. Tissue Microarrays, Immunohistochemical Methods and Scoring

Tissue microarrays (TMAs) were constructed using duplicate tumor cores from primary tumors and an automated tissue microarrayer (VTA-100, Veridiam, San Diego, CA, USA). Tissue sections from the TMAs were used for PD-L1 immunohistochemistry (IHC) using the Ventana autostainer system according to manufacturer’s protocols. Positivity was defined by the presence of any single cell with membranous expression of PD-L1 either in tumor or in immune cells (total cells). In addition, whole tissue sections (4 μm) were prepared for a subset of patients in this cohort based on the expression of PD-L1 in tumor cells (positive and randomly selected negative cases) and stained using an anti-phosphorylated STAT3 (pSTAT3) antibody (Y705). At least 300 tumor cells were counted in five different high power fields in order to calculate the percentage of pSTAT3 positive cells. The median percentage of expression was used as a cut-off for dividing patient tumors in pSTAT3-high and pSTAT3-low expressing ones. Furthermore, cell pellets from cell lines were collected, fixed in formalin and embedded in paraffin to prepare cell blocks. Subsequently, IHC was performed using anti-pSTAT3 and anti-PD-L1 antibodies, as previously described [41]. The antibodies used are listed in Table S2. Moreover, Ki67 immunohistochemical staining and evaluation method have been previously described [42].

### 4.5. Fluorescence in Situ Hybridization

The *PDL1* gene locus was analyzed on formalin-fixed paraffin-embedded (FFPE) cell pellets by Fluorescence in situ hybridization using the probe and protocols recommended by the manufacturer (ZytoVision GmbH, Bremerhaven, Germany).

### 4.6. Transient Transfections and Gene Silencing

Cells were seeded at a density of 0.2–0.6 × 10^6^ cells/ml 24 h before transfection. Silencing of breast cancer cell line BT549 with siRNA oligonucleotides specific for the gene sequence of *STAT3* was carried out with Lipofectamine 2000 (Thermofisher, Waltham, MA, USA) reagent according to the company’s protocol. Approximately 3 × 10^6^ cells were transfected with 300 nM specific siRNA or Control siRNA (All Stars Negative, QIAGEN, Hilden, Germany). Whole-cell lysates were prepared 48 h after transfection. The siRNA oligonucleotides or plasmids with which cells were transfected are listed in Table S3.

### 4.7. Proliferation Assay

Mouse 4T1-shCTR and 4T1-sh*Stat3* tumor cells were seeded at a density of 4 × 10^3^ cells per well containing 100 uL in 96-well plates with complete RPMI-1640 medium for three days. For each cell line and each time point (0, 24, 48 and 72 h) six replicates were used. A mixture of XTT labeling with electron-coupling reagents was then made according to manufacturer’s protocol (Cell Proliferation Kit II (XTT), Roche, Basel, Switzerland). For each time point, measurement of cell viability was performed via microplate reader at a wavelength of 490 nm (reference wavelength: 650 nm).

### 4.8. Lentiviral Vectors

Tumor cells (4T1) were transduced with lentiviral vectors encoding shRNA for murine *Stat3* or corresponding control vector (listed in Table S3) as previously described [43].

### 4.9. 4T1 Breast Cancer Animal Model and Tumor Growth Assessment

For the in vivo tumor model, 2 × 10^5^ 4T1 cells (shCTR and sh*Stat3*) in a volume of 50 mL PBS were injected into the mammary fat pad of anesthetized four to six week-old female BALB/c mice, purchased from Charles River Laboratory. Mice were sacrificed three weeks after tumor cell injection, and tumors were weighed after dissection. Tumor size was measured externally using calipers, and tumor volumes were estimated using the following equation: V = 4/3π × (*d*/2)^2^ × D/2, where *d* is the minor tumor axis and D is the major tumor axis. All ethical permits were obtained from the Swedish Board of Agriculture (N95/15).

### 4.10. Tumor Dissociation

Tumors were cut into smaller pieces using scalpels. Dissected tumors were minced in dissociation buffer (TrypLe and Stem Cell Pro Accutase (Life Technologies, Waltham, MA, USA); 1:1) and incubated at 37 °C for 30 mins in order single-cell suspensions to be obtained. Thereafter, suspensions were passed through a 19 G syringe needle, filtered and washed in PBS (10% FBS) to be used for flow cytometry.

### 4.11. Flow Cytometry

To prevent antibody nonspecific binding, single-cell suspensions of tumors were pre-incubated with anti-CD16/32 mAb (BioLegend, San Diego, CA, USA) on ice for 15 minutes before a 30-minute incubation on ice with specific antibodies. Cells were stained using antibodies for extracellular markers. T-regulatory (FoxP3^+^) cells were stained as well according to manufacturer’s instruction (BD Biosciences, San Jose, CA, USA). The viability of cells was verified using 7AAD or the Live/Dead fixable dead cell stain (Life Technologies, Waltham, MA, USA). Samples were acquired with a LSR II (BD Biosciences, San Jose, CA, USA) and analyzed using FlowJo software (Tree Star, Ashland, OR, USA) [15]. All antibodies used are listed in Table S2 and gating strategies are depicted in Supplementary Figures S5 and S6.

### 4.12. Lung Metastasis Colony Assay

Lungs from tumor-bearing mice were dissociated to single cells in an enzymatic buffer containing RPMI (Gibco/Life technologies, Waltham, MA, USA) 5% FBS (Gibco/Life technologies, Waltham, MA, USA) 0.2 mg/mL collagenase IV (Life technologies, Waltham, MA, USA) 0.2 mg/mL dispase (Life technologies, Waltham, MA, USA) and 0.1 mg/mL DNAse I (Sigma-Aldrich, St. Louis, MO, USA) as previously described [44,45]. Cell suspensions were plated in the presence of 60 uM 6-thioguanine (Sigma-Aldrich, St. Louis, MO, USA). Tumor cells resistant to 6-thioguanine were allowed to form colonies for approximately 10 days and then fixed with methanol, stained with crystal violet, and counted under a dissection microscope.

### 4.13. Immunofluorescence for Macrophage Markers in Human Breast Tumors

Paraffin-embedded breast cancer patient samples were cut in 4 μm thick whole-tissue sections and treated for antigen retrieval with sodium citrate buffer (Biocare Medical). All tumor sections were blocked in blocking buffer containing PBS (Sigma, St. Louis, MO, USA), 0.3% Triton X-100 (Sigma), 10 % fetal bovine serum (Gibco, Waltham, MA, USA) and 1% Bovine Serum Albumin (Sigma) and immunostained with the appropriate antibodies: anti-CD163 (Leica, Wetzlar, Germany) and anti-CD11c (Leica) and all secondary antibodies were conjugated with AlexaFluor 488 or AlexaFluor 546 fluorochromes (Life Technologies, Waltham, MA, USA). Cell nuclei were labeled with DAPI (Invitrogen Corp., Carlsbad, CA, USA). Isotype specific antibodies were used to ensure specificity of the antibodies. All antibodies used are listed in Table S2. Eight independent fields from each tumor section were analyzed by using LSM T-PMT Zeiss confocal microscope and quantified by ImageJ software (NIH, Bethesda, MD, USA).

### 4.14. Patient Cohorts

The patient cohort used in the study consists of women diagnosed with primary breast cancer between 1997 and 2005 in Stockholm health care region who were retrospectively selected using the Stockholm-Gotland Breast Cancer Registry. Data on clinical and pathological tumor characteristics, survival, loco-regional and systemic treatments, and follow-up have been collected and reported elsewhere [42]. The Cancer Genome Atlas (TCGA) provisional dataset [46], including 1081 patients with primary breast cancer, was used as a validation cohort. Available RNA-sequencing data were retrieved from cBioportal [47,48].

### 4.15. Gene Expression Profiling Using Microarrays

Gene expression profiling has been performed from all primary tumors in the cohort. Details regarding experimental methods have been previously described [42] and the gene expression microarray data can be accessed at the Gene Expression Omnibus database under accession number GSE48091. All ethical permits were obtained from Karolinska Institutet’s (Stockholm, Sweden) ethics committee (Dnr 2006/1183-31/2, 2016/1505-32). According to the ethics board, no additional informed consent from the patients was required for these analyses. 

### 4.16. Preprocessing and Normalization of the Microarray Gene Expression Data

The preprocessing and normalization of the microarray gene expression data were performed within R computing environment. Specifically, the raw data were background corrected, normalized and summarized to obtain a log-transformed expression value for each probe set using the RMA method [49] implemented in the aroma.affymetrix R package [50]. A nonspecific filter was employed and probe sets with the highest interquartile range were kept in the case of multiple mappings to the same Entrez Gene ID.

### 4.17. Phosphorylated STAT3-Associated Gene Signature

The phosphorylated STAT3-associated gene signature (pSTAT3-GS) was applied to the patient cohort as described in the original publication [51]. Specifically, the provided pSTAT3-GS’s gene symbols were converted to Entrez Gene IDs using DAVID’s Gene ID Conversion Tool [52] (DAVID Bioinformatics Resources version 6.8), and mapped to the microarray’s probe sets. The (continuous) pSTAT3-GS scores, i.e., signed averages, were computed using the “sig.score” function from the genefu R package [53] (R package version 2.14.0). In total, 114 (out of the 123) pSTAT3-GS’s genes were mapped and therefore used in the signature scores’ calculation. For the TCGA dataset, in total 122 genes of the pSTAT3-GS were mapped. 

### 4.18. Statistical Analyses 

Statistical analyses and graphical representations regarding the in vitro and in vivo models were performed using GraphPad Prism software version 7.0 (GraphPad Software Inc., San Diego, CA, USA) while analyses concerning patient material were performed within R computing environment version 3.5.1. Wilcoxon–Mann–Whitney test, Student’s t-test and two-way ANOVA were used as indicated. Spearman’s rank correlation coefficient was used to evaluate the associations between continuous variables and Fisher’s exact test for the associations between categorical variables. A *p*-value equal to or less than 0.05 was considered as statistically significant.

## 5. Conclusions 

In this study, STAT3-mediated regulation of PD-L1 and modulation of immune microenvironment were shown in breast cancer. More research is warranted towards the further characterization of TME interactions and anti-tumor immunity, thus providing breast cancer patients better therapeutic options and prognostication factors.

## Supplementary Materials

The following are available online at https://www.mdpi.com/2072-6694/11/10/1479/s1, Table S1. List of cancer cell lines used in the study; Table S2. List of antibodies used in the study; Table S3. List of primers, siRNA and shRNA target sequences used in the study; Table S4. Correlation of PD-L1 protein expression in tumor, immune and total cells with pSTAT3 protein expression in human breast cancer patients; Table S5. Correlation of PD-L1 protein expression in tumor cells with grade and Ki67 status in human breast cancer patients; Figure S1. Expression of PD-L1 and genetic alteration in control cell line; Figure S2. Correlation of PD-L1 transcript with pSTAT3-GS score and expression patterns in TCGA Provisional database. Figure S3. Expression of pSTAT3 protein in breast cancer patients; Figure S4. Correlations of CD163^+^ cell and CD163^+^ CD11c^+^ cell percentage with proliferation status (Ki67) and tumor grade in human breast cancer patients. Figure S5. Gating strategies for macrophage panel MHC class II. Figure S6. Gating strategies for lymphocytic panel.
