# Supplementary material for: STAT3 Activity Promotes Programmed-Death Ligand 1 Expression and Suppresses Immune Responses in Breast Cancer
Source: Cancers (Basel). 2019 Oct 1;11(10):1479. doi: 10.3390/cancers11101479 (PMC6827034; doi:10.3390/cancers11101479)

cancers MDPI

Article

STAT3 Activity Promotes Programmed-Death Ligand 1 Expression and Suppresses Immune Responses in Breast Cancer

Ioannis Zerdes [1,*], Majken Wallerius [1], Emmanouil G. Sifakis [1], Tatjana Wallmann [1], Stina Betts [1], Margarita Bartish [1], Nikolaos Tsesmetzis [2], Nicholas P. Tobin [1], Christos Coucoravas [3], Jonas Bergh [1,4], George Z. Rassidakis [1,5], Charlotte Rolny [1] and Theodoros Foukakis [1,4,*]

Table S1. List of cancer cell lines used in the study.

Cell Line	Tissue	Organism	Disease	Comments
MCF7[1]	Mammary gland/breast	Human	Adenocarcinoma	Luminal
MDA-MB-231 [1]	Mammary gland/breast	Human	Adenocarcinoma	Triple-negative
BT549 [1]	Mammary gland/breast	Human	Ductal carcinoma	Triple-negative
SKRB3[2]	Mammary gland/breast	Human	Adenocarcinoma	ER-/PR-/HER2+
4T1 [3]	Mammary gland/breast	Mouse	Animal stage IV human breast cancer	Originated from tumors developed in BALB/cfC3H mouse; highly metastatic; Triple—negative
MAC2A [4]	Lymphoid	Human	Anaplastic large cell lymphoma	ALK—negative
HDLM2 [5]	Lymphoid	Human	Hodgkin lymphoma	-

[1] Purchased from Sigma-Aldrich and ATCC, USA; [2] Provided by Sarah Walker and David Frank (Department of Medical Oncology, Dana-Farber Cancer Institute, and Department of Medicine, Harvard Medical School, Boston, MA, USA); [3] Purchased from Karmanos Cancer Institute, Wayne State University, USA; [4.] A gift from Dr. Marshal Kadin (Boston, MA, USA); [5] Purchased from DSMZ, Germany; **Abbreviations:** ER, estrogen receptor; PR, progesterone receptor; HER2, human epidermal growth factor receptor 2; ALK,Anaplastic Lymphoma Kinase.

Table S2. List of antibodies used in the study.

Antibody	Cat. no	Species	Company	Method(s)
STAT3	#4904	Rabbit (79D7)	Cell Signaling Technology	Western blot
pSTAT3 (Y705)	sc-8059	Mouse (B-7)	Santa Cruz Biotechnology	Western blot, IHC
pSTAT3 (Y705)	#9145	Rabbit (D3A7)	Cell Signaling Technology	Western blot, IHC
PD-L1	#13684	Rabbit (E1L3N)	Cell Signaling Technology	Western blot, IHC
PD-L1	#740-4859	Rabbit (clone SP263)	Ventana (Roche)	IHC
b-actin	A5441	Mouse	Sigma-Aldrich	Western blot
Anti-rabbit IgG	#7074	Goat	Cell Signaling Technology	Western blot

(HRP-linked)				
Anti-mouse IgG (HRP-linked)	#7076	Horse	Cell Signaling Technology	Western blot
Anti-biotin (HRP-linked)	#7075	Goat	Cell Signaling Technology	Western blot

Antibodies	Company	Method
CD163	Leica	Immunofluorescence
CD11c		
AlexaFluor 488	Life Technologies	
AlexaFluor 546		
DAPI	Invitrogen Corp	

Antibodies	Company	Method
CD11b (M1/70)	BD Bioscience	Flow cytometry
CD45 (30-F11)		
Ly6C (AL-21)		
Ly6G (1A8)		
CD86 (GL1)		
CD11c (HL3)		
CD3 (500A2/145-2c11)		
CD8 (53-6.7)		
CD4 (RM4-5)		
CD69 (H1.2F3)		
CD49b (HM ALPHA2)		
MHC class II (I-A/I-E)		
MHC class I (H-2K(d))		
CD25 (PC61)		
FoxP3 (MF23)		
F4/80 (BM8)	BioLegend	Flow cytometry
MRC1 (C068C2)		
CD8a (53-6.7)		
CD279 (29F.1A12)		
CD274 (10F.9G2)		

Table S3. List of primers, siRNA and shRNA target sequences used in the study.

Gene	Sequence (5′- 3′)	Company	Method
***PD-L1* (human)**	Forward: CTCCAAATGAAAGGACTCAC<br>Reverse: TCCCTTTTTCTTAAACGGAAG	Sigma-Aldrich	RT-qPCR
**b-actin (human)**	Forward: GACGACATGGAGAAAATCTG<br>Reverse: ATGATCTGGGTCATCTTCTC	Sigma-Aldrich	RT-qPCR
***pd-l1* (mouse)**	Forward: CAAGTGAGAATGCTAGATGTG<br>Reverse: TCCATCTTGAGTCTTTGGAC	Sigma-Aldrich	RT-qPCR
***18S rRNA* (mouse)**	Forward: TTCCTTACCTGGTTGATCCTGCC<br>Reverse: AGCCATTCGCAGTTTCACTGTAC	Sigma-Aldrich	RT-qPCR
**siRNA *STAT3* (human)**	GAGAUUGACCAGCAGUAUA	Dharmacon™ (Lafayette, CO, USA)	Transient transfection
**shRNA *Stat3* (mouse) MISSION® Plasmid**	NM_011486.3.1238s1c1<br>CCGGCCTGAGTTGAATTATCAGCTTCTCGAGAAGCTGATAATTCAACTCAGGTTTTTG	Sigma-Aldrich	Lentiviral vector trandsduction

Abbreviations: siRNA, small interfering RNA; shRNA, small hairpin RNA; RT-qPCR, real-time quantitative polymerase chain reaction.

Table S4. Correlation of PD-L1 protein expression in tumor, immune and total cells* with pSTAT3 protein expression in human breast cancer patients. Fisher's exact test was used for the statistical analyses. (* either tumor or immune cells).

	**PD-L1 IHC Expression in Tumor Cells**			**PD-L1 IHC Expression in Immune Cells**			**PD-L1 IHC Expression in Total Cells**		
	**Positive**	**Negative**	***p*-Value**	**Positive**	**Negative**	***p*-Value**	**Positive**	**Negative**	***p*-Value**
pSTAT3-high ($n = 41$)	22	19	0.66	24	17	0.08	26	15	0.19
pSTAT3-low ($n = 42$)	20	22		16	26		20	22	

Table S5. Correlation of PD-L1 protein expression in tumor cells with grade and Ki67 status in human breast cancer patients.

	**PD-L1 IHC expression in tumor cells**		
	**Positive ($n = 23$)**	**Negative ($n = 22$)**	***p*-value***
**Grade 1-2**	4	18	1.982e-05
**Grade 3**	19	4	
**Ki67 < 16%**	2	12	0.00106
**Ki67 >= 16%**	20	9	

* Fisher's exact test. For Ki67, there was one patient with unknown status.

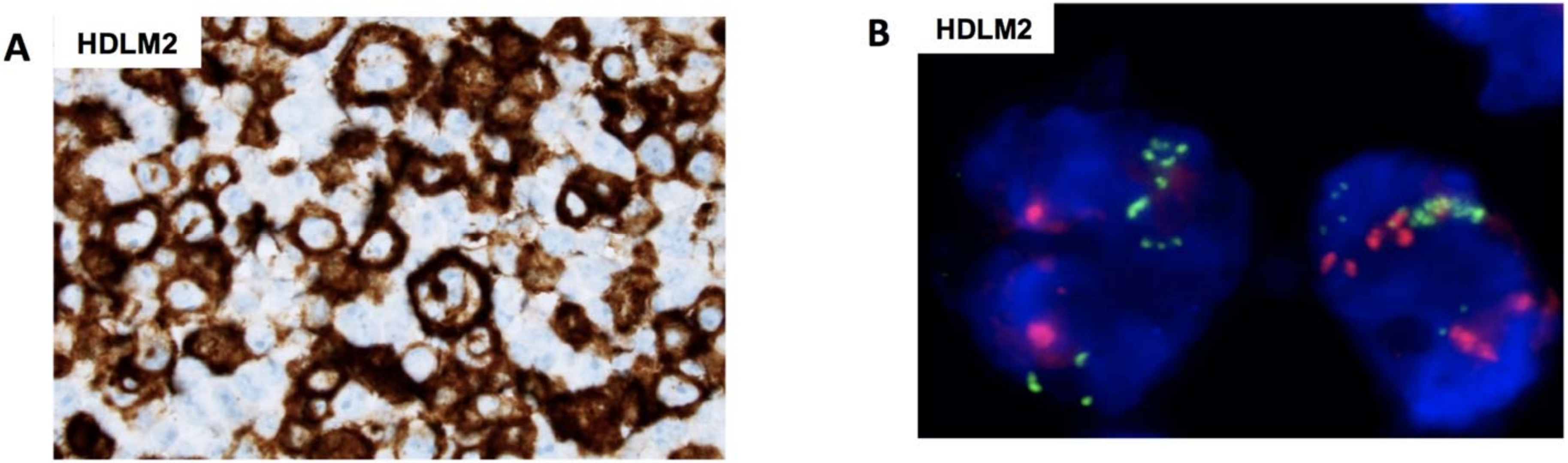

Figure S1. Expression of PD-L1 and genetic alteration in control cell line. A. High PD-L1 protein expression in immunohistochemistry on sections of FFPE cell pellets of HDML2 Hodgkin lymphoma cell line (used as positive control). Original magnification: ×200. **B.** Fluorescence in situ hybridization analysis for PD-L1 probe performed on HDLM2 cell blocks. The validated probe (green signal) cover the gene locus at 9p24.1. A centromeric chromosome 9 probe (CEN9, red signal) was used as a control. HDLM2 Hodgkin lymphoma cell line was used as positive control *PD-L1* gene amplification. Original magnification: ×630.

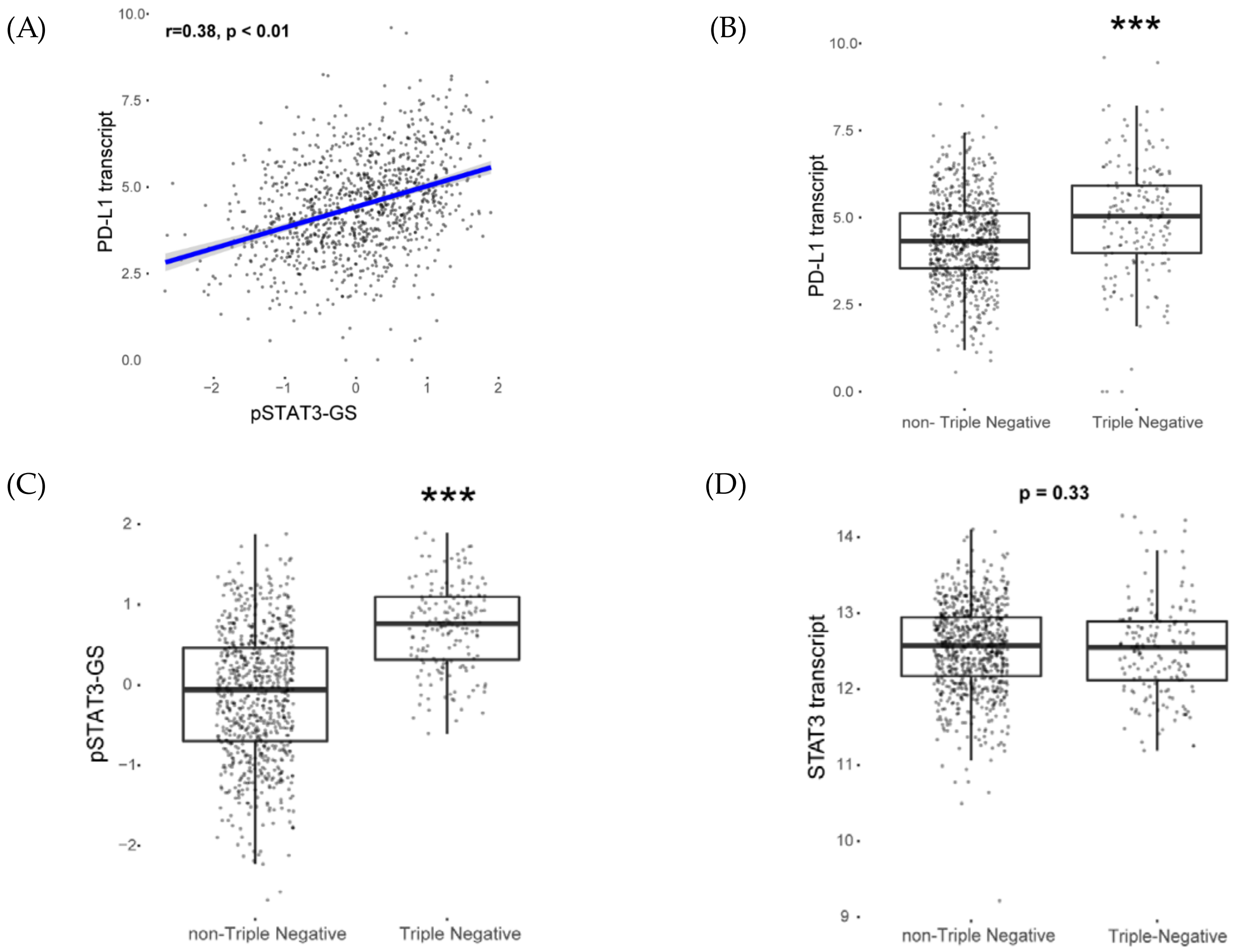

Figure S2. Correlation of PD-L1 transcript with pSTAT3-GS score (**A**) and expression patterns in TCGA Provisional database. Expression patterns of PD-L1 transcript (**B**), pYSTAT3-GS score (**C**) and STAT3 transcript (**D**) in triple-negative (TN) versus non-TN early breast cancer patients in TCGA Wilcoxon–Mann–Whitney test and Spearman's rank correlation coefficient were used (* $p < 0.05$; **, $p < 0.01$; *** $p < 0.001$).

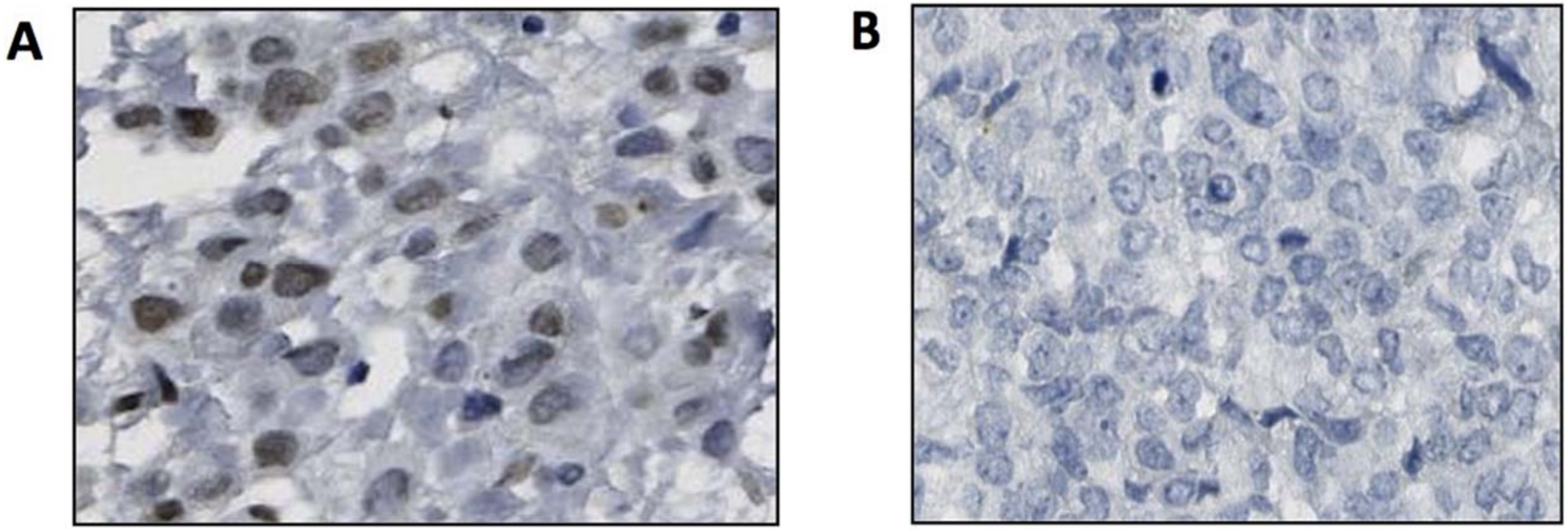

Figure S3. Expression of pSTAT3 protein in breast cancer patients. Immunohistochemical staining for pSTAT3 (Y705) protein was performed in a subset of patients (n = 83). Representative photos of A. high pSTAT3 protein expression in tumor cells and B. no pSTAT3 protein expression on whole tissue sections of FFPE breast cancer patient tumors. Original magnification: ×400.

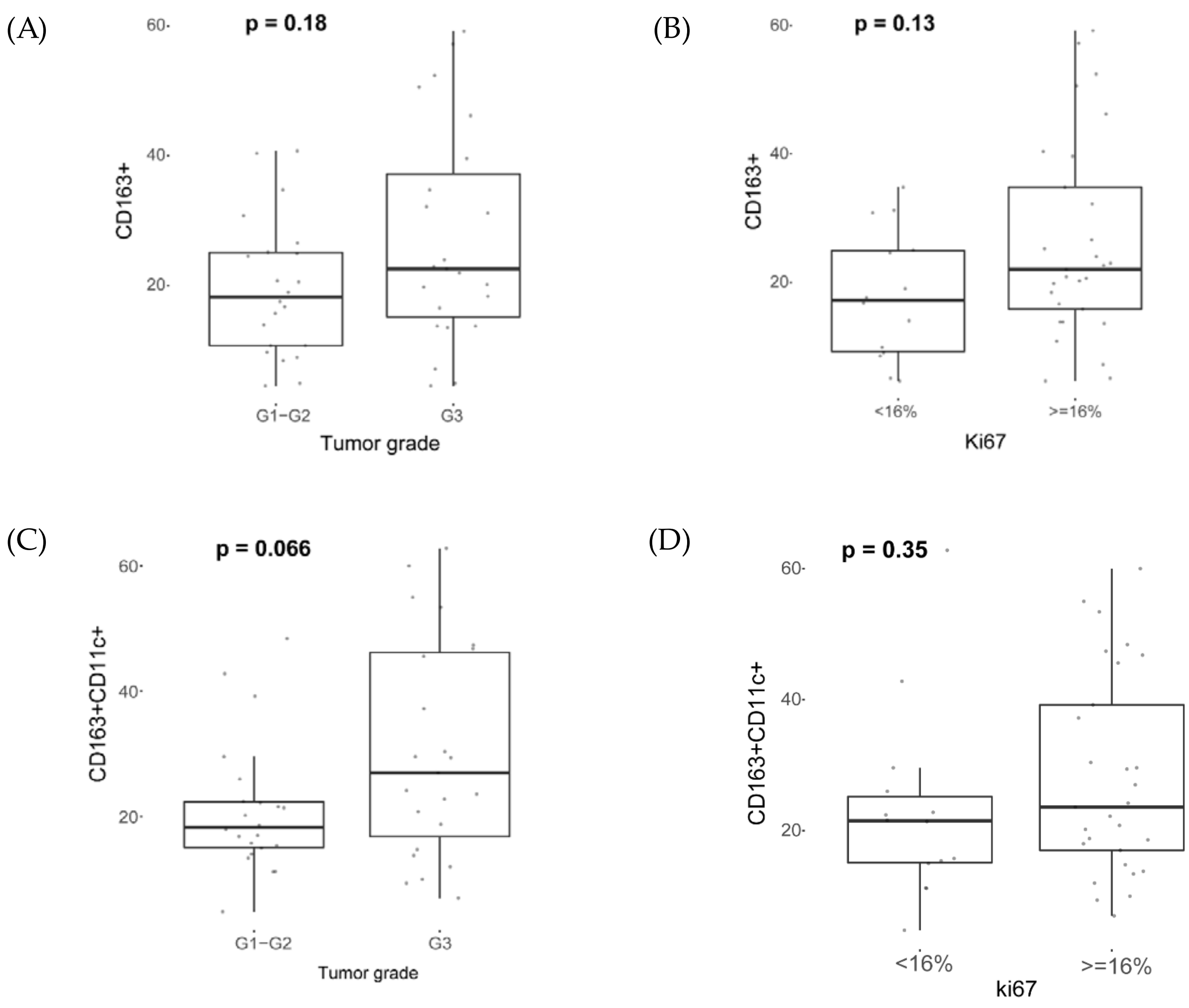

Figure S4. Correlations of CD163+ cell percentage (**A**,**B**) and CD163+ CD11c+ cell percentage (**C**,**D**) with tumor grade and proliferation status (Ki67) in human breast cancer patients. Wilcoxon–Mann–Whitney test was used (* $p < 0.05$; ** $p < 0.01$; *** $p < 0.001$).

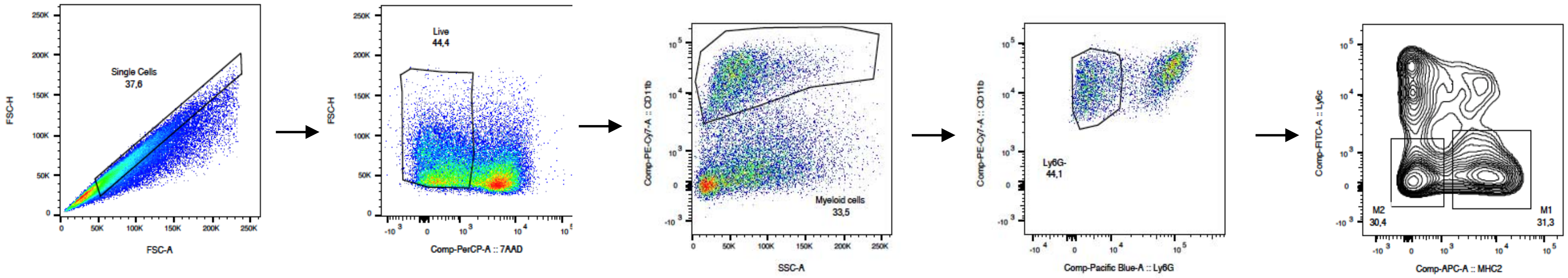

Figure S5. Gating strategies for macrophage panel MHC class II.

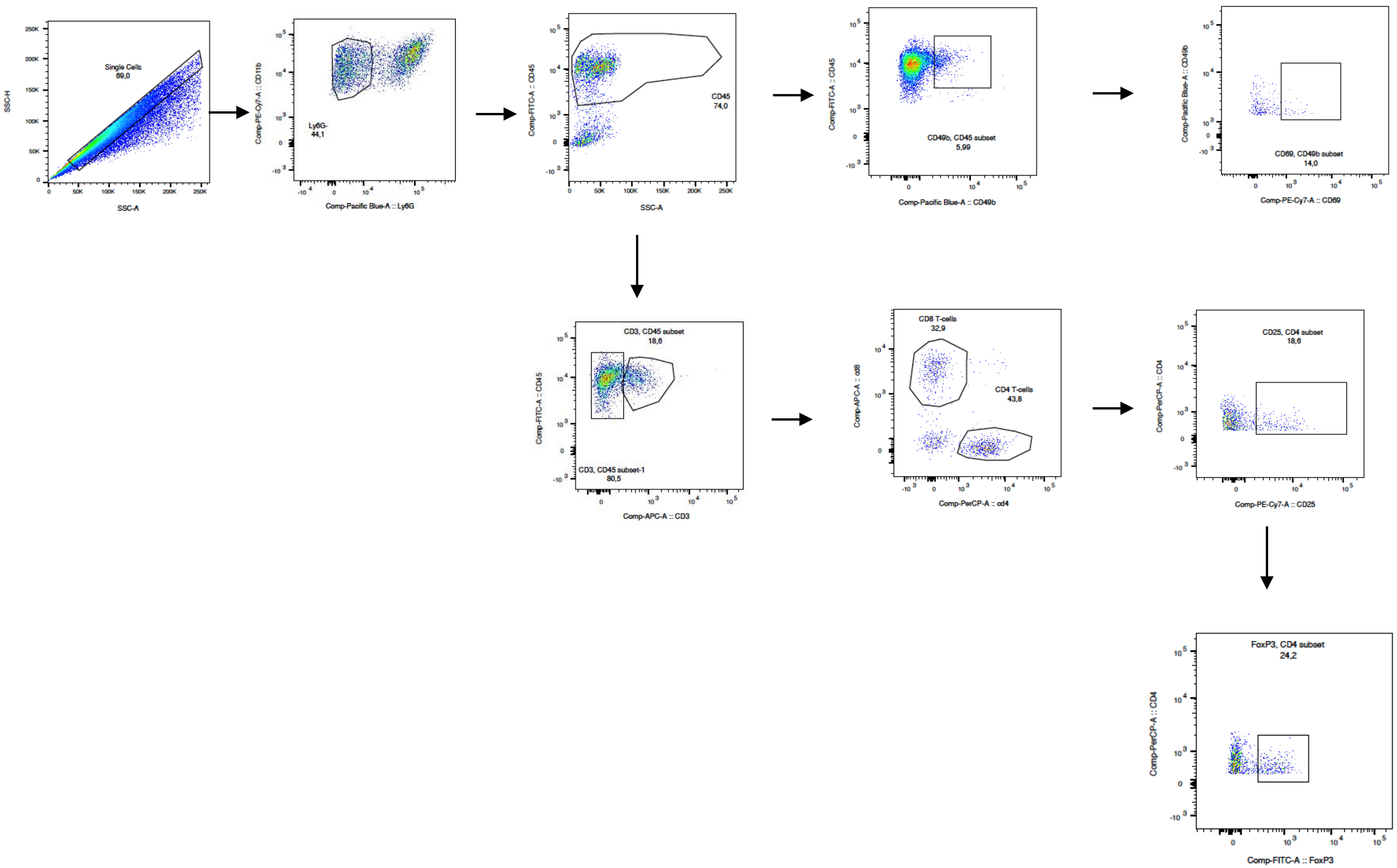

Figure S6. Gating strategies for lymphocytic panel.

Western blots corresponding to Fig. 1A

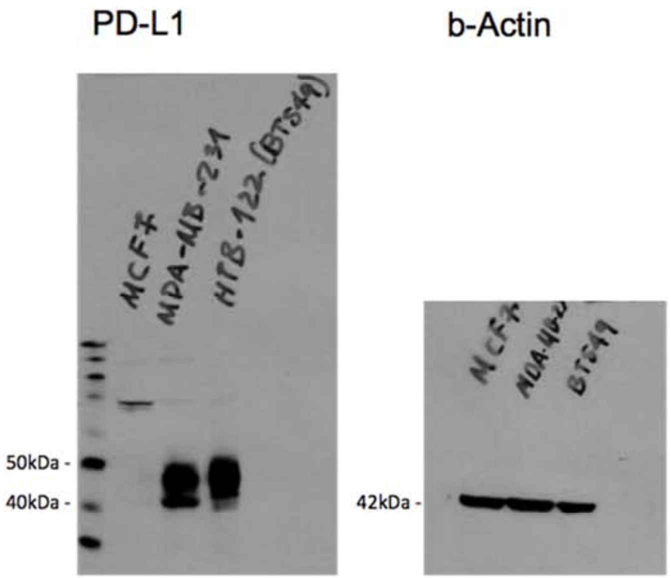

Western blots corresponding to Fig. 1B

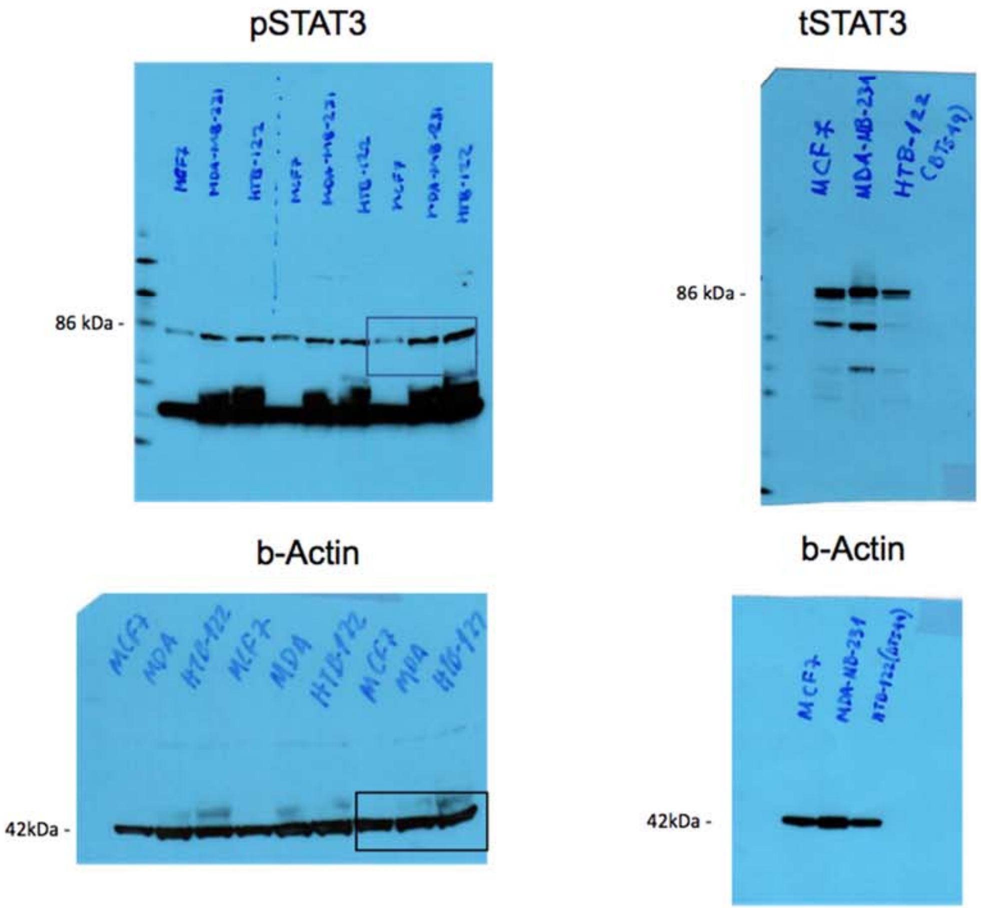

Western blots corresponding to Fig. 2D

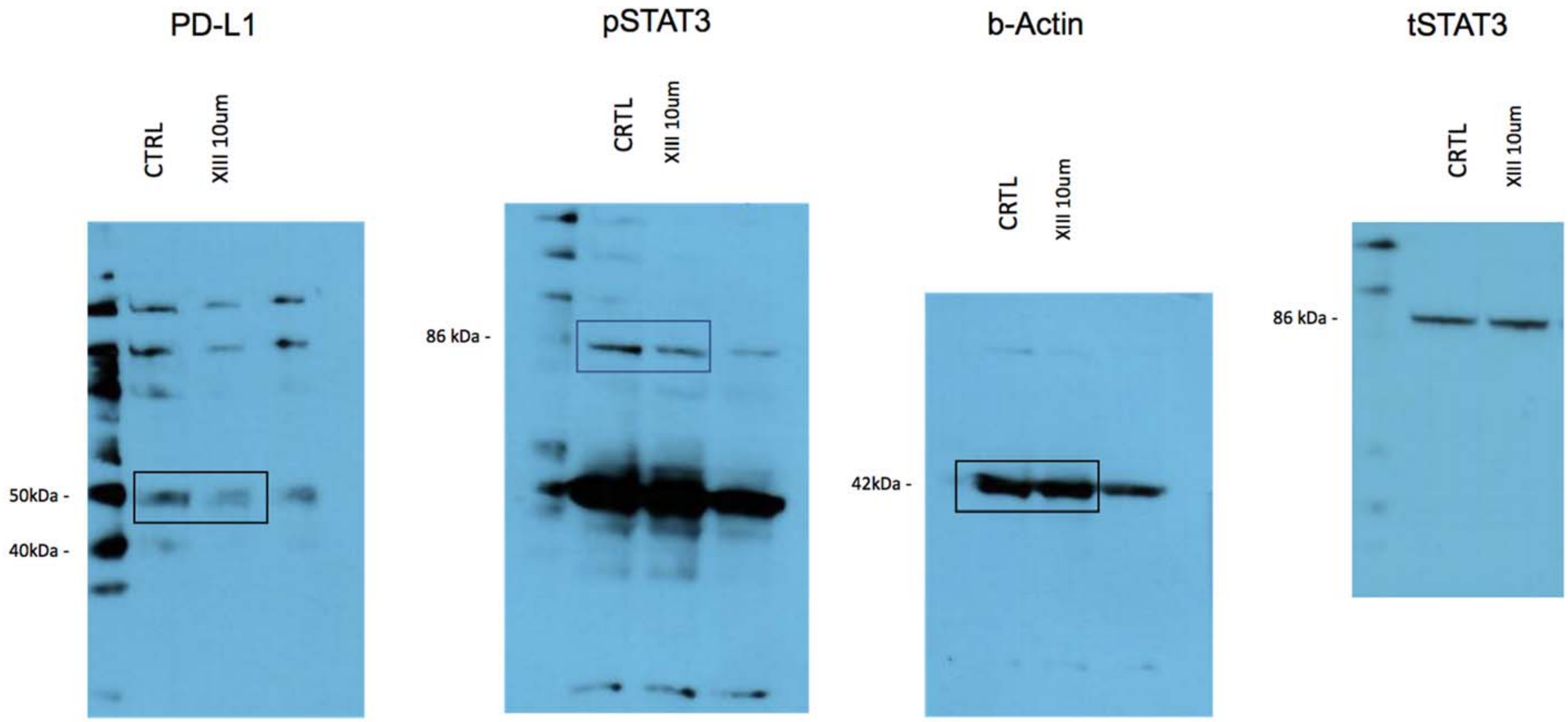

Western blots corresponding to Fig. 2E

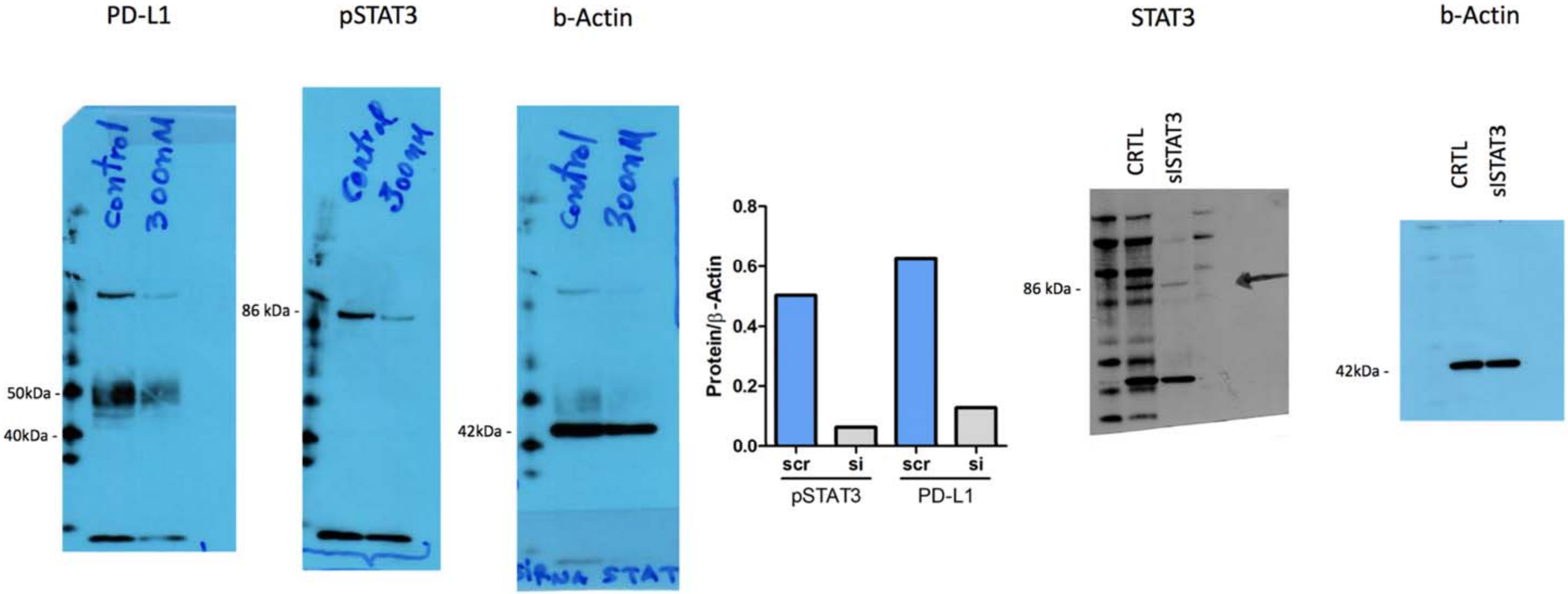

Western blots corresponding to Fig. 2F

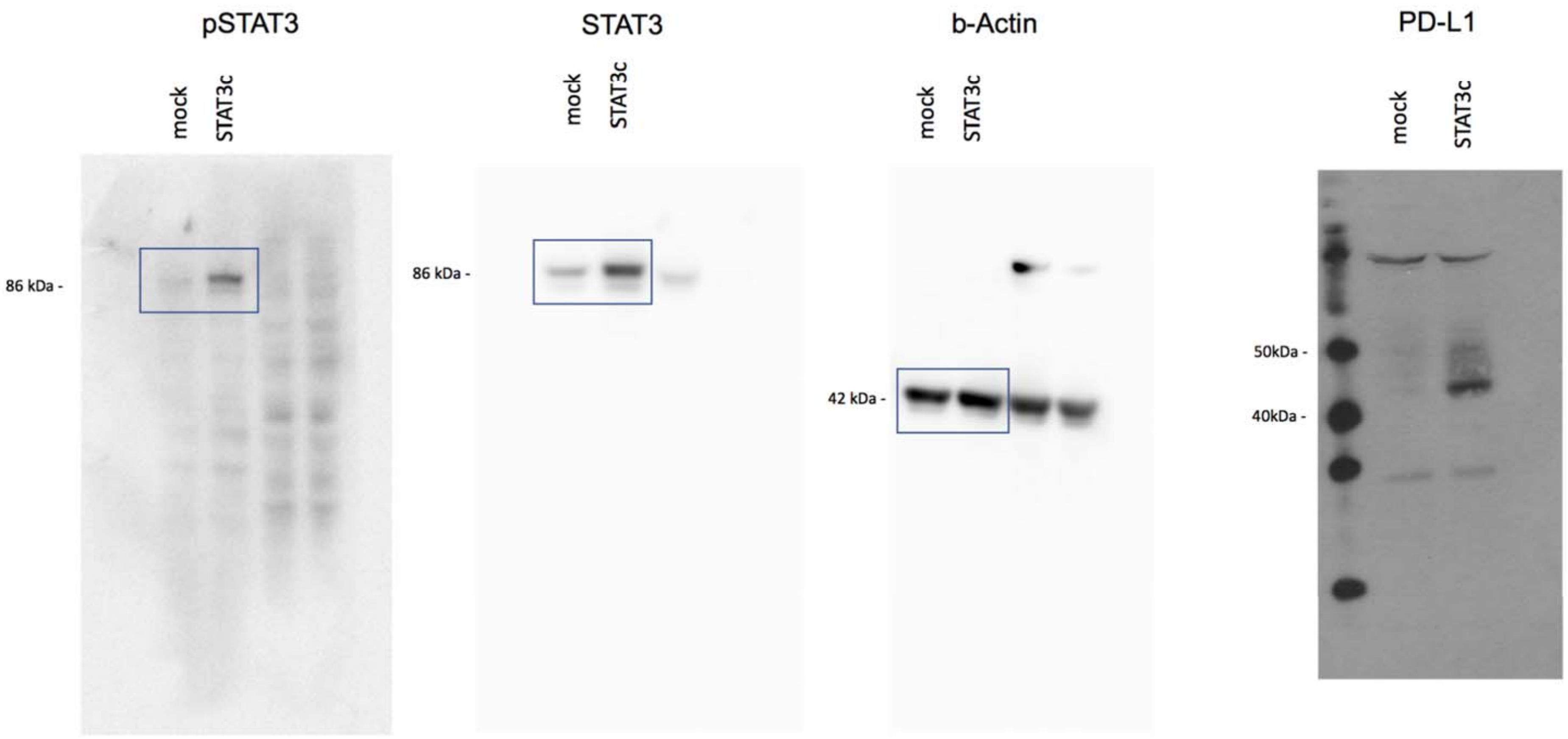

Western blots corresponding to Fig. 2H

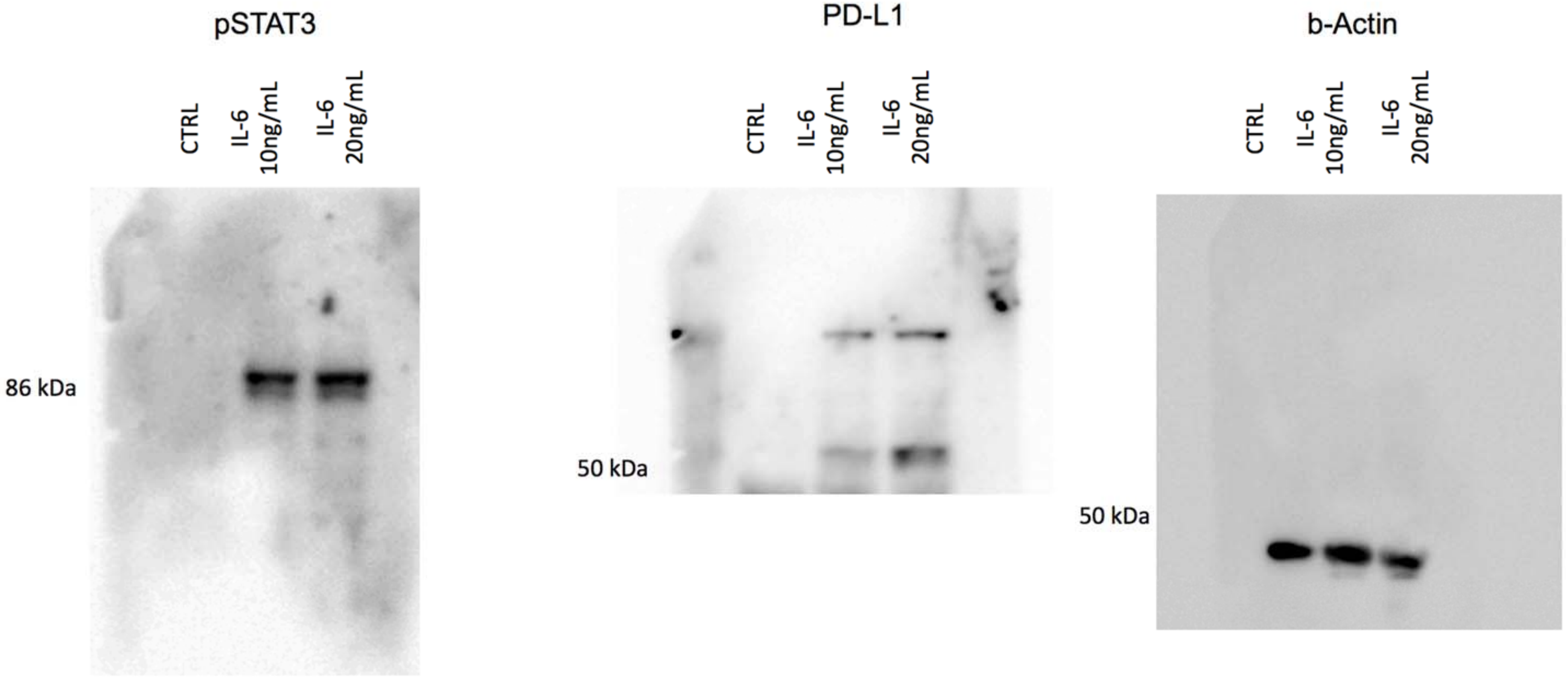

Western blots corresponding to Fig. 3B

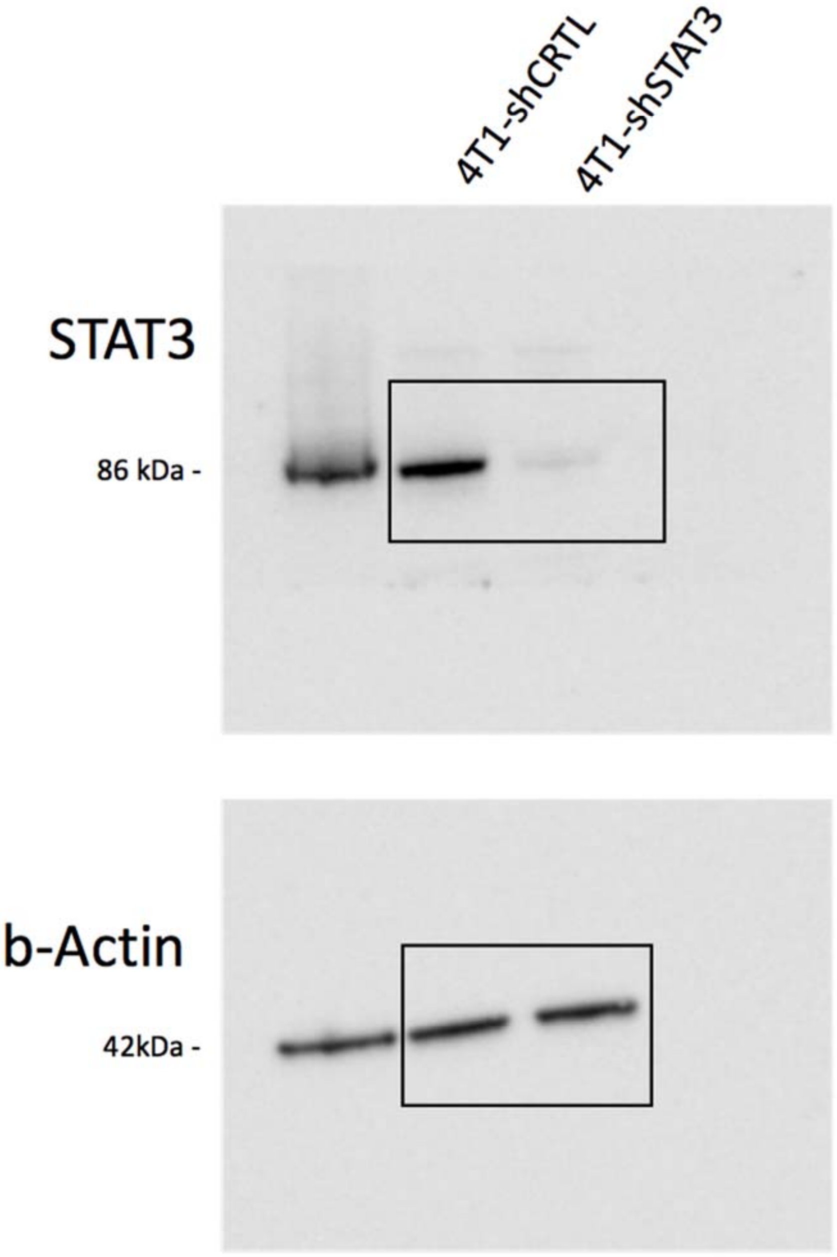

Supplement: Supplementary file 1 [file cancers-11-01479-s001.pdf]
